# Licensed Practical Nurses in Team Triage: A Safe Way to Address Nursing Shortages in the Emergency Department

**DOI:** 10.7759/cureus.40926

**Published:** 2023-06-25

**Authors:** Samita M Heslin, Candice King, Sarah Williams, Alison Rowe, Mariel Kasschau, Brian McMahon, Eric J Morley

**Affiliations:** 1 Emergency Medicine, Stony Brook University Hospital, Stony Brook, USA

**Keywords:** registered nurse shortage, licensed practical nurse, specimen mislabeling, emergency department, team triage

## Abstract

Background

There is a Registered Nurse (RN) shortage across the United States that is predicted to intensify in the upcoming years. RNs are an integral part of Emergency Departments (EDs) and perform many vital tasks, including IV placement, blood draws, medication administration, acute assessments, and patient hand-offs. Thus, RN staffing is a crucial part of ED operations, and ED initiatives should account for RN workforce shortages. Given the increase in ED visits and crowding, throughput initiatives that can expedite patient care are integral to the functioning of an ED. Team Triage is a throughput initiative that has been shown to improve ED time to provider, length of stay, and left without being seen rates. In our institution, we created a Team Triage model where advanced practice providers (APPs) perform a patient’s initial evaluation in triage and place orders for labs, intravenous (IV) catheters, and imaging. Given the RN staffing shortage, we incorporated Licensed Practical Nurses (LPNs) in Team Triage to place IV catheters and draw blood work for laboratory tests. The objective of this investigation was to describe a Team Triage model that incorporated LPNs and to report the patient safety and productivity of this model.

Methods

This was a single-site retrospective study at a large, academic, tertiary care center with over 100,000 annual visits. Adult patients who self-presented to the ED and went through Team Triage (11 am-11 pm) between Jan 1, 2020, and Jan 31, 2020, were included in this study. LPNs staffed the Team Triage, along with APPs. LPNs placed IV catheters and drew blood specimens for the Team Triage patients. The primary outcomes studied were the proportion of specimens mislabeled by LPNs, the proportion of patients receiving IV catheters, the proportion of patients receiving blood work, blood tubes drawn per hour, and IVs inserted per hour in Team Triage.

Results

During the study period, 1355 patients went through Team Triage. Of these patients, 1075 (79%) were ordered for blood work, and 1017 (75%) were ordered for an IV catheter. All Team Triage blood work and IV catheter placements were completed by LPNs, who staffed 372 hours of Team Triage. A total of 2558 blood tubes were collected by LPNs. The LPNs cared for 2.9 patients per hour, collected 6.9 blood tubes per hour, inserted 2.7 IV catheters per hour, and collected 2.4 blood tubes per patient. The LPNs had a 0% specimen mislabeling rate.

Conclusion

Due to the significant RN workforce shortage impacting Emergency Medicine coupled with increased ED crowding, there is a significant need to evaluate the integration of LPNs into Team Triage to place IV catheters and perform blood draws. This study shows that incorporating LPNs in Team Triage is a productive and safe way to address nursing shortages in Emergency Medicine.

## Introduction

There is a nationwide nursing shortage impacting many specialties, including Emergency Medicine, in the United States. In 2021 alone, the total supply of Registered Nurses (RNs) decreased by over 100,000 more than any other year in the last four decades [1]. By 2030, it is predicted there will be a total national deficit of nearly one million RN jobs [2]. Emergency Departments (EDs) play an important role in the health of our nation, and Emergency RNs play a critical role in the delivery of emergency medical care. Emergency RNs often care for multiple ED patients at once and perform a significant number of tasks, including intravenous (IV) catheter placement, blood draws, medication administration, acute assessments, patient hand-offs, and more. RN staffing is a crucial part of ED operations, and ED workflows should account for RN staffing and possible shortages.

With increasing ED visits and crowding [3], incorporating throughput initiatives to expedite the care of patients is imperative. At our institution, we created a new intervention called Team Triage to facilitate the care of patients arriving in the ED [4]. After the traditional triage is completed with vital signs, a chief complaint with associated symptoms, and an Emergency Severity Index (ESI) level assigned, patients are seen by Team Triage. In Team Triage, a nurse practitioner or physician assistant (advanced practice provider [APP]) performs a comprehensive initial evaluation and places orders for laboratory tests, peripheral IV catheter placement, and imaging. Due to the RN staffing shortage, we collaborated with Licensed Practical Nurses (LPNs) to place IVs and draw blood work for laboratory tests as part of the Team Triage initiative.

Many EDs have attempted Team Triage models to reduce wait times, improve the length of stay, and decrease left without-being-seen rates [5-7]. Although there are many variations on how Team Triage is staffed, most incorporate an RN [8-10]. A great need remains to assess the integration of LPNs in Team Triage, especially in the current landscape of significant RN staffing shortages.

Goals of this investigation

The objective of this investigation was to describe a Team Triage model that incorporated LPNs and to report the patient safety of this model based on the proportion of specimens mislabeled and the productivity of this model based on patients per hour, blood tubes drawn per hour, and IVs inserted per hour.

## Materials and methods

Study design

This was a single-site, retrospective study investigating regularly collected ED operations data. The LPN’s Team Triage intervention and the subsequent data gathered were part of ED quality improvement initiatives. This project was submitted to the Institutional Review Board and was approved as a quality improvement study.

Setting

This study occurred in a large, suburban, academic, tertiary care university hospital with an annual ED volume of over 100,000 visits, of which 75% are adults. The ED is separated into four zones: two adult acute care zones, one adult critical care zone, and one pediatric zone. The ED is staffed with attending Emergency Medicine physicians who provide 100% coverage in all zones. Additionally, nurse practitioners, physician assistants, and resident physicians work with the attending physicians in each zone. There are three main entrances to the ED: a main entrance where adult patients self-present, an entrance where pediatric patients self-present during designated hours, and an entrance where patients (adult and pediatric) are present by ambulance. Each of these areas is staffed by an RN who performs the traditional triage. The Team Triage room is positioned near the main adult entrance in a 20 ft x 20 ft room adjacent to the traditional triage room.

Selection of participants

ED patient data were collected from the Cerner (North Kansas City, MO) electronic health record. There were no missing data. All patients who went through Team Triage between 1/1/2020 and 1/31/2020 were studied.

Interventions

All patients who arrive in the ED go through traditional triage, where an RN ascertains the reason for the visit, performs vital signs, collects relevant past medical history, documents current medications, and assigns an ESI level. Adult patients who arrive through the main entrance between 11 am and 11 pm also go through Team Triage after the traditional nurse triage. The Team Triage room is adjacent to the traditional triage room and is staffed by an LPN and an APP for 12 hours a day (11 am and 11 pm), seven days a week.

**Figure 1:**
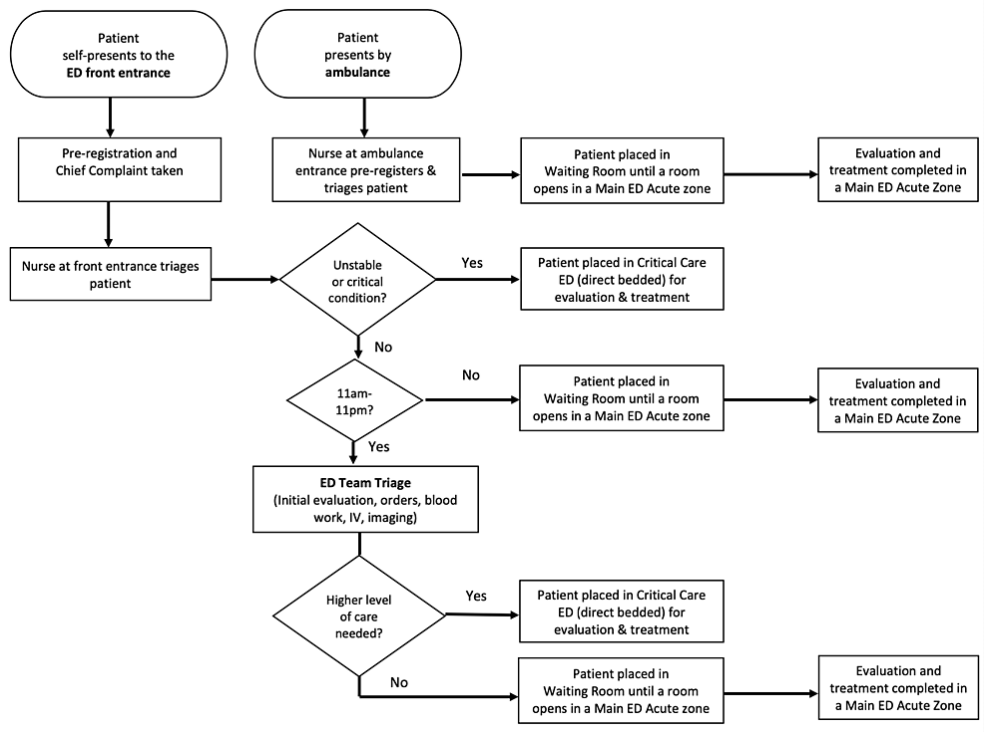
Flow of the Team Triage process IV: Intravenous catheter; ED: Emergency department

Once the traditional triage is completed, patients with symptoms concerning a time-sensitive critical condition (e.g., cerebrovascular accident, heart attack) or unstable vital signs are brought immediately to the critical care zone and directly bedded. Patients without concerns for high-acuity illnesses proceed to the Team Triage. Figure 1 details the patient flow through Team Triage. Once in the Team Triage room, an initial evaluation is performed by the APP, who takes a history and performs a focused physical exam. The APP then places orders to facilitate the initial workup, including orders for IV catheter placement, laboratory tests, and radiology imaging. The LPN reviews the orders and performs the tasks that are within their scope of practice, including the placement of IVs and blood draws. After the Team Triage evaluation and any procedures that need to be completed (i.e., blood work, IV catheter placement), the patient is placed back into a waiting location until an exam room opens in the main ED. 

If an IV and a laboratory test are both ordered, the LPN collects the blood samples at the time of IV cannulations. The IV catheter is secured and remains in the patient until they are discharged. If only laboratory tests are ordered, the LPN collects the blood samples by using a butterfly needle, which is removed immediately after blood sample collection. Before either the IV catheter or butterfly needle cannulation, the vein is first located by visualization and palpation. Then, the skin is cleaned with chlorhexidine. The IV catheter (BD, Franklin Lakes, NJ) size is generally 18 gauge or 20 gauge. The butterfly needle (BD, Franklin Lakes, NJ) size is 21 or 23 gauge.

Several types of blood tubes are drawn by LPNs in Team Triage based on the laboratory orders placed by the APP. In our institution, the lavender top blood tube is used to collect blood samples for the Complete Blood Count (CBC); the mint top blood tube for chemistry; the grey top blood tube for the lactate; the pink top blood tube for the blood type and screens; and the blue top blood tube for the coagulation factors.

Once the blood tubes are drawn, they are labeled with a patient label. This occurs after the LPN verifies the patient's identity on the label using two patient identifiers, such as the patient’s name and date of birth. The labeled blood tubes are then placed into a plastic bag, and another patient label is placed on the outside of the plastic bag. The plastic bag is placed into a pneumatic tube and sent from the ED to the laboratory through the hospital’s pneumatic tube system. The type and screen (pink blood tube) are the only blood tubes that also need to be accompanied by a printed requisition. This tube must be signed by the phlebotomist along with the ordered requisition from the APP.

Specimens are considered mislabeled if they arrive at the lab with the wrong patient label, have no patient label on the blood tube, have no patient label on the plastic bag, or if the order requisition (for a type and screen blood tube) is not present or not signed. Mislabeled specimens are discarded by the laboratory and are required to be redrawn.

All LPNs receive training in both onboarding orientations and yearly refreshers about the policies and procedures for venous cannulation, IV catheter placement, specimen collection, and specimen labeling. The education includes didactic teaching through lectures and simulations with a manikin arm, where the LPN practices IV cannulation and catheter placement, specimen collection, patient label verification with two patient identifiers, and specimen labeling.

## Results

During the study period, 1355 patients went through Team Triage. Of these patients, 1075 (79%) were ordered for blood work, and 1017 (75%) were ordered for an IV catheter. There were also a total of 2558 blood tubes collected. All blood specimens collected and all IV catheters placed were performed by LPNs, who staffed 372 hours in Team Triage during the study period (Table 1).

**Table 1:** Characteristics of Team Triage during the study period LPN: License practical nurse; IV: Intravenous

Team Triage metric	#
Patients who went through Team Triage	1355
Hours of LPN coverage in Team Triage	372
Patients who had blood drawn in Team Triage	1075
Blood tubes collected by LPNs in Team Triage	2558
IV catheters inserted by LPNs in Team Triage	1017

For the patients who received blood work by LPNs in Team Triage, the average age was 46 (SD 18), 38% were male, the median ESI was three (IQR 3-3), and 78% were discharged from the ED (Table 2).

**Table 2:** Characteristics of the patients who had blood drawn in Team Triage during the study period SD: Standard deviation; ESI: Emergency severity index; IQR: Interquartile range; ED: Emergency department

Demographic	
Mean Age (SD)	46 (18)
Sex (% Male)	38%
Median ESI (IQR)	3 (3-3)
Discharged from the ED	78%

The LPNs cared for 2.9 patients per hour, collected 6.9 blood tubes per hour, and inserted 2.7 IV catheters per hour. The LPNs collected 2.4 blood tubes per patient and mislabeled zero specimens (Table 3).

**Table 3:** Productivity and patient safety metrics for LPNs in Team Triage LPN: Licensed practical nurse; IV: Intravenous

LPN productivity or patient safety metric	
Patients seen per hour by LPN	2.9
Blood tubes collected per hour by LPN	6.9
IV catheters inserted per hour by LPN	2.7
Blood tubes collected per patient	2.4
Specimens mislabeled	0

Table 3: Productivity of LPNs in Team Triage. LPN: Licensed practical nurse; IV: Intravenous

**Figure 2:**
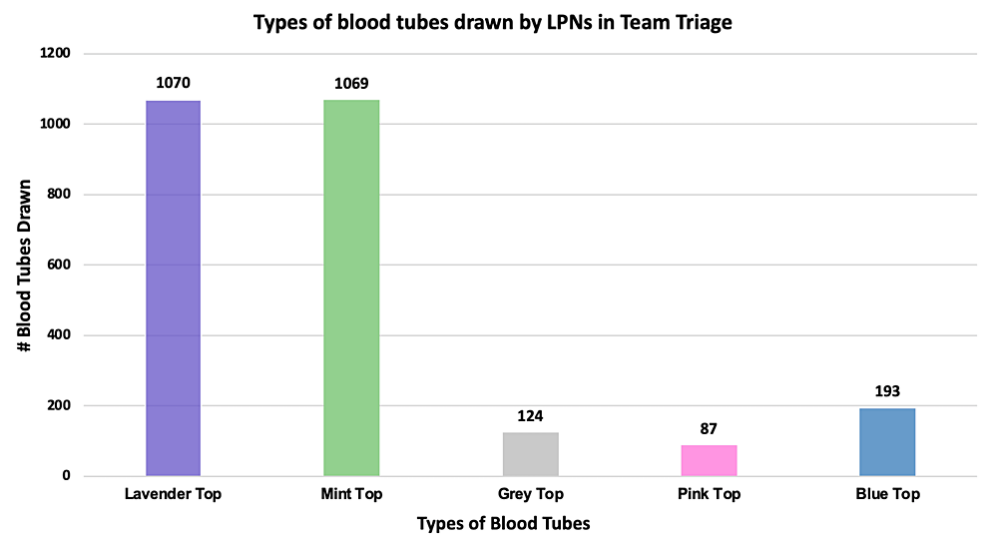
Types of blood tubes drawn by LPNs in Team Triage during the study period

Of the 2558 blood tubes collected, 1070 (42%) were for the CBC (lavender top blood tube); 1069 (42%) were for the chemistry (mint top); 124 (4.9%) were for the lactate (grey top); 87 (3.4%) were for the blood type and screen (pink top); and 193 (7.5%) were for the coagulation factors (blue top). The distribution of the types of blood tubes collected is shown in Figure 2.

## Discussion

Team Triage has been shown to improve door-to-provider time, reduce ED length of stay, and decrease left without being seen rates [11-13]. Despite the many variations of Team Triage, most models incorporate an RN for IV catheter placement and blood draws. RNs are critical to the functioning of an ED and perform many important tasks, including ESI-level assignments, patient assessments, and medication administration. However, given the widespread RN workforce shortage, there is a significant need to study the incorporation of LPNs in the Team Triage process for IV catheter insertion and blood specimen collection.

This study shows that LPNs can be successfully integrated into Team Triage and are productive members of the team. LPNs in this study performed blood draws on 2.9 patients per hour, collected 6.9 blood tubes per hour, inserted 2.7 IV catheters per hour, and collected 2.4 blood tubes per patient. Placing IV catheters along with drawing and sending blood work to Team Triage has positive downstream effects. When Team Triage patients are assigned an exam room in the main ED, their RNs do not need to place an IV and can focus on other aspects of patient care. In our experience, this has a positive impact on RN satisfaction. Additionally, laboratory testing results sent in Team Triage are in-process or completed by the time the patient is assigned an exam room in the main ED, which can expedite the patient’s workup, treatment, and disposition.

Specimen mislabeling rates are an additional important patient safety metric to consider. Specimen mislabeling has a significant impact on patients and the healthcare system. Identification errors may result in delays in care, unnecessary treatment, and transfusion reactions [14]. Previous studies have shown that over half of identification errors were due to primary specimen labeling errors, and one out of 18 identification errors resulted in adverse events [15]. In this study, the LPNs in Team Triage had a 0% specimen mislabeling rate.

This study has several limitations. It was a single-center study at a large, suburban, academic ED and may not be generalizable to other settings. This study also has a retrospective design and may have biases and limitations intrinsic to the study design. The study was performed over a one-month period, but there is no evidence to suggest that a longer or different time period would have significantly impacted the study results. The study was only conducted on self-presenting adult patients; further studies would be needed to assess its applicability to pediatric patients and patients presenting by ambulance. Finally, the LPN scope of practice may vary by state and institution. Future initiatives should validate the findings from this study in other ED settings and models of Team Triage.

## Conclusions

There is currently a significant RN workforce shortage impacting Emergency Medicine. RNs are an integral part of the functioning of an ED, and operational or workflow changes should consider RN staffing and shortages. Team Triage has been shown to improve ED throughput and typically incorporates an RN. Our ED implemented a Team Triage model, during which patients in the waiting room receive a comprehensive initial evaluation and orders from an APP. Due to the RN staffing shortage, we incorporated LPNs into Team Triage to place IV catheters and draw blood work for laboratory tests. This study shows that LPNs were productive members of Team Triage and had a 0% specimen mislabeling rate. LPNs have an important role in the ED Team and can safely help with IV catheter placement and blood draws, which can improve ED throughput and flow.
